# American Medical Association

**Published:** 1868

**Authors:** W. B. Atkinson


### AMERICAN MEDICAL ASSOCIATION.

The Nineteenth Annual Meeting of the American Medical Association will be held in Washington on Tuesday, May 5th, 1868, at 11 o'clock A. M.

The following Committees are expected to report:

On Ophthalmology—Dr. Jos. S. Hildreth, Illinois, Chairman.

On Cultivation of the Cinchona Tree—Dr. J. M. Toner, D. C., Chairman.

On Surgical Diseases of Women—Dr. Theophilus Parvin, Indiana, Chairman.

On Rank of Medical Men in the Navy—Dr. N. S. Davis, Illinois, Chairman.

On Insanity—Dr. C. A. Lee, N. Y., Chairman.

On American Medical Necrology—Dr. C. C. Cox, Maryland, Chairman.

On Leakage of Gas Pipes—Dr. J. C. Draper, New York, Chairman.

On Medical Ethics, ———, Chairman.

On Plan of Organization—Dr. C. C. Cox, Maryland, Chairman.

On Provision for the Insane—Dr. C. A. Lee, New York, Chairman.

On the Climatology and Epidemics of Maine—Dr. J. C. Weston.

Of New Hampshire, Dr. P. A. Stackpole.
Vermont, Dr. Henry Janes.
Massachusetts, Dr. Alfred C. Garratt.
Rhode Island, Dr. C. W. Parsons.
Connecticut, Dr. E. K. Hunt.
New York, Dr. W. F. Thoms.
New Jersey, Dr. Ezra M. Hunt.
Pennsylvania, Dr. D. F. Condie.
Maryland, Dr. O. S. Mahon.
Georgia, Dr. Juriah Harriss.
Missouri, Dr. Geo. Engelman.
Alabama, Dr. R. Miller.
Texas, Dr. T. J. Heard.
Illinois, Dr. R. C. Hamil.
Indiana, Dr. J. F. Hibberd.
District of Columbia, Dr. T. Antisell.
Iowa, Dr. J. W. H. Baker.
Michigan, Dr. Ab'm. Sager.
Ohio, Dr. J. W. Russell.
California, Dr. F. W. Hatch.
Tennessee, Dr. Joseph Jones.
West Virginia, Dr. E. A. Hildreth.
Minnesota, Dr. Samuel Willey.

On Clinical Thermometry in Diphtheria—Dr. Joseph G. Richardson, New York, Chairman.

On the Treatment of Disease by Atomized Substances—Dr. A. G. Field, Iowa, Chairman.

On the Ligation of Arteries—Dr. Benj. Howard, New York, Chairman.

On the Treatment of Club-Foot without Tenotomy—Dr. L. A. Sayre, New York, Chairman.

On the Radical Cure of Hernia—Dr. G. C. Blackman, Ohio, Chairman.

On Operations for Hare-Lip—Dr. Hammer, Missouri, Chairman.

On Errors of Diagnosis in Abdominal Tumors—Dr. G. C. E. Weber, Ohio, Chairman.

On Prize Essays—Dr. Charles Woodward, Ohio, Chairman.

On Medical Education—Dr. A. B. Palmer, Michigan, Chairman.

On Medical Literature—Dr. Geo. Mendenhall, Ohio, Chairman.

Secretaries of all medical organizations are requested to forward lists of their Delegates, as soon as elected, to the Permanent Secretary.

W. B. ATKINSON.

